# Supplementary material for: Lipid-siRNA Conjugates Targeting High PD-L1 Expression as Potential Novel Immune Checkpoint Inhibitors
Source: Biomolecules. 2025 Feb 15;15(2):293. doi: 10.3390/biom15020293 (PMC11852376; doi:10.3390/biom15020293)
Supplement: Supplementary file 1 [file biomolecules-15-00293-s001.zip › biomolecules-3462134-supplementary.pdf]

Supporting Material

Lipid-siRNA Conjugates Targeting High PD-L1 Expression as Potential Novel Immune Checkpoint Inhibitors

Rina Tansou [1], Takanori Kubo [1*], Haruka Nishida [1], Yoshio Nishimura [2], Keichiro Mihara [3], Kazuyoshi Yanagihara [1,4], and Toshio Seyama [1]

[1]Laboratory of Molecular Cell Biology, Department of Life Science, Faculty of Pharmacy, Yasuda Women's University, Hiroshima 731-0153, Japan

[2]School of Pharmaceutical Sciences, Ohu University, Fukushima 963-8611, Japan

[3]Department of the International Center for Cell and Gene Therapy, Fujita Health University, Toyoake 470-1192, Japan

[4]Division of Rare Cancer Research, National Cancer Center Research Institute, Tokyo 104-0045, Japan

* Corresponding Author

Takanori Kubo, E-mail: kubo-t@yasuda-u.ac.jp

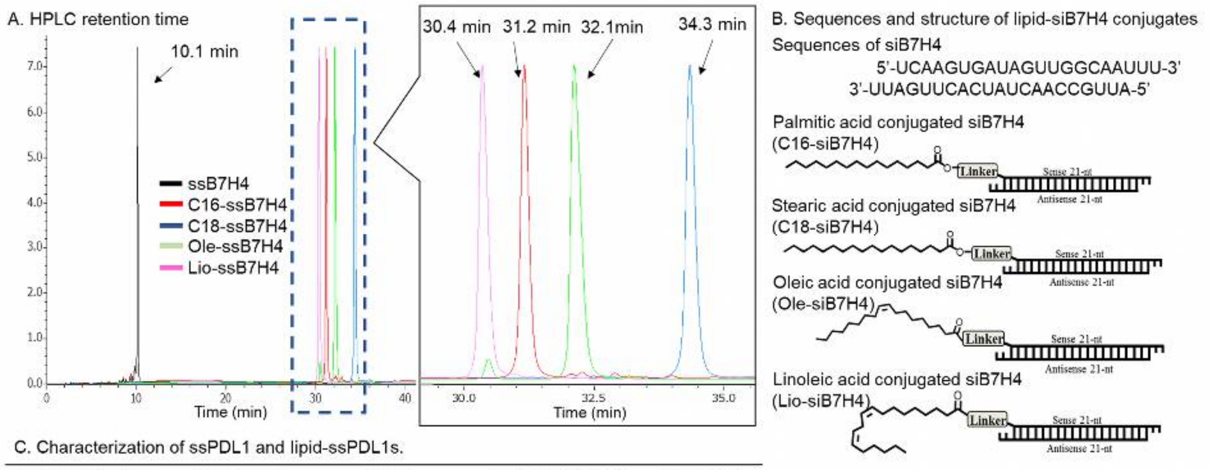

C. Characterization of ssPDL1 and lipid-ssPDL1s.

Conjugate ssRNA	Conjugated fatty acid	HPLC retention time[a]	MALDI-TOF MS[b] Found / Calcd	Yield[c] (%)
ssB7H4	-----	10.1	6699.8 / 6699.0	-----[d]
C16-ssB7H4	Palmitic acid	31.2	7116.4 / 7116.6	38.5
C18-ssB7H4	Stearic acid	34.3	7145.6 / 7144.6	49.4
Ole-ssB7H4	Oleic acid	32.1	7143.8 / 7142.6	69.6
Lio-ssB7H4	Linoleic acid	30.4	7141.1 / 7140.6	53.7

[a] Linear gradient of CH_3CN at concentrations varying from 7 to 70% over 40 min in 100 mM TEAA (pH 7.0) using an octadecylsilyl column.
[b] A saturated solution of 2,4,6-trihydroxyacetophenone in 50 mg/mL diammonium hydrogen citrate in 50% acetonitrile was used as the matrix.
[c] Overall product yields were determined by measuring the absorbance at 260 nm after HPLC purification.
[d] Purified ssRNA was purchased.

Figure S1. Sequences and characterization of lipid-ssB7H4s and lipid-siB7H4s.

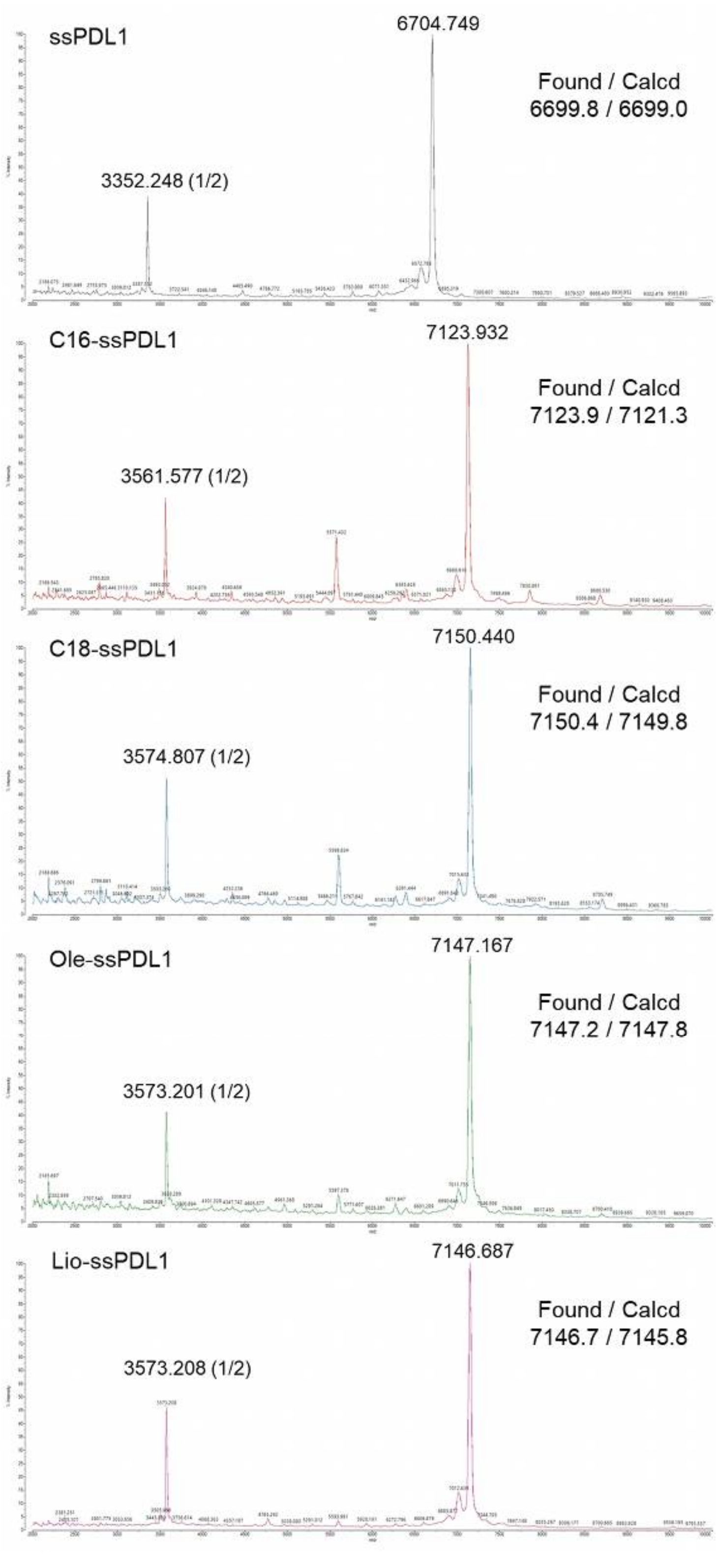

Figure S2A. MALDI TOF-MS analysis of ssPDL1 and lipid-ssPDL1

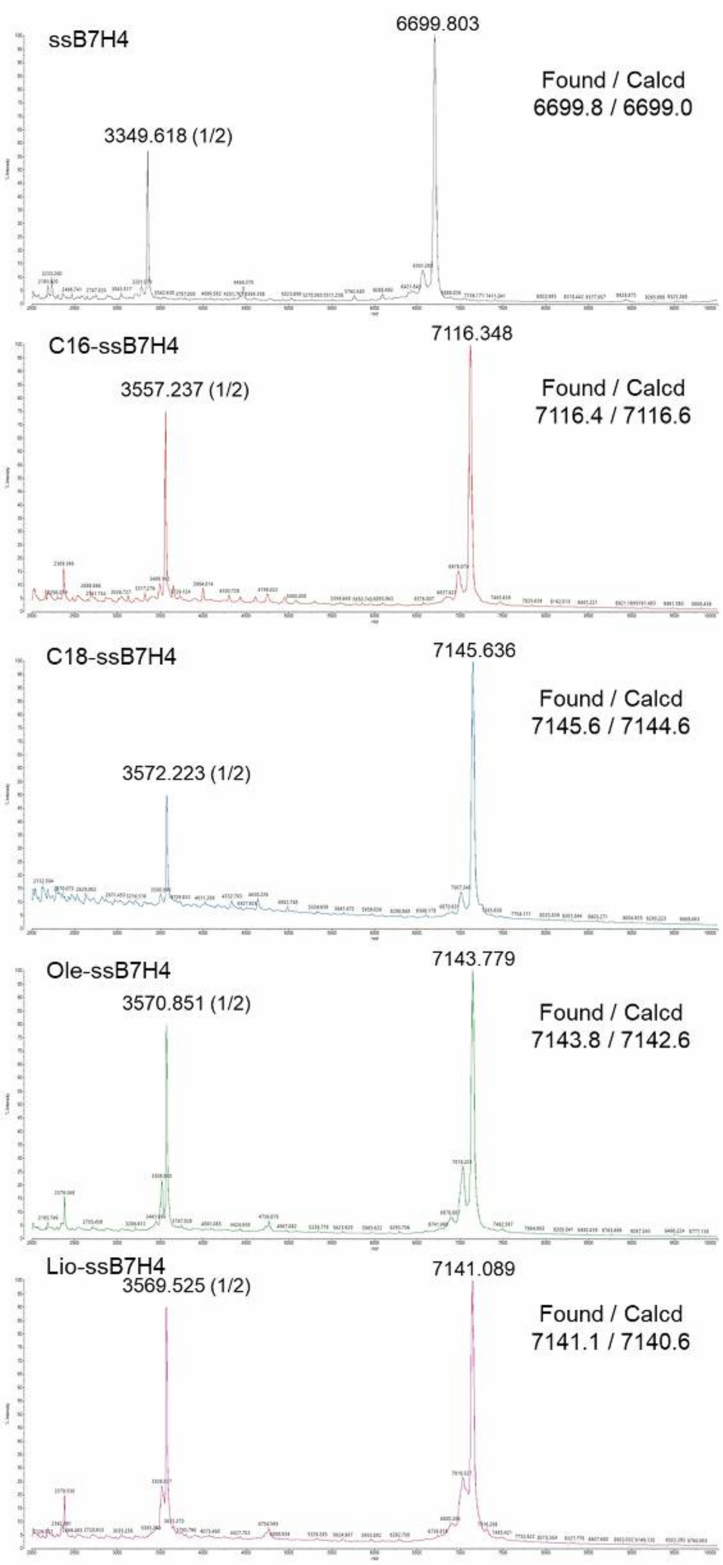

Figure S2B. MALDI TOF-MS analysis of ssPDL1 and lipid-ssPDL1

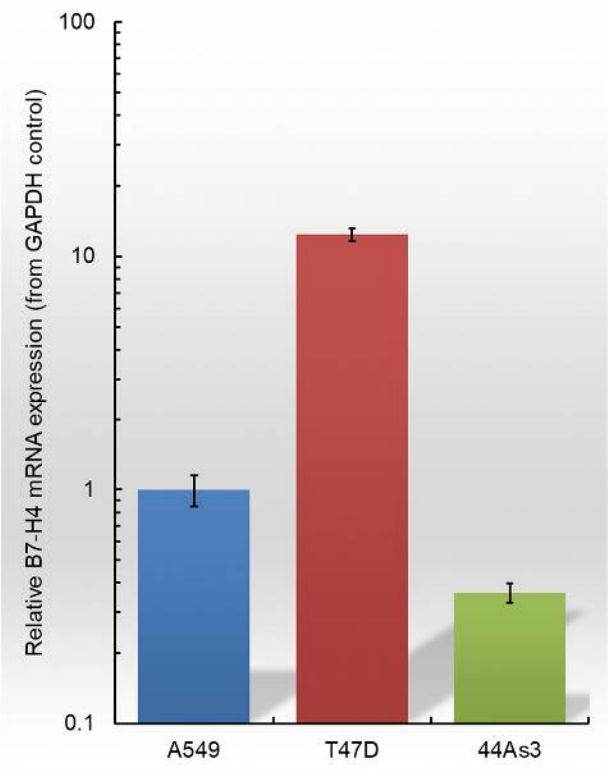

Figure S3. Relative B7-H4 expression in A549, T47D, and 44As3 cells. Expression levels of B7-H4 mRNA were detected in A549 cells and compared with those in T47D and 44As3 cell lines using reverse transcription-quantitative PCR (RT-qPCR). Cancer cell lines exhibit differential expression of B7H4. Comparing the three cancer cell lines, T47D cells exhibit the highest B7-H4 expression.

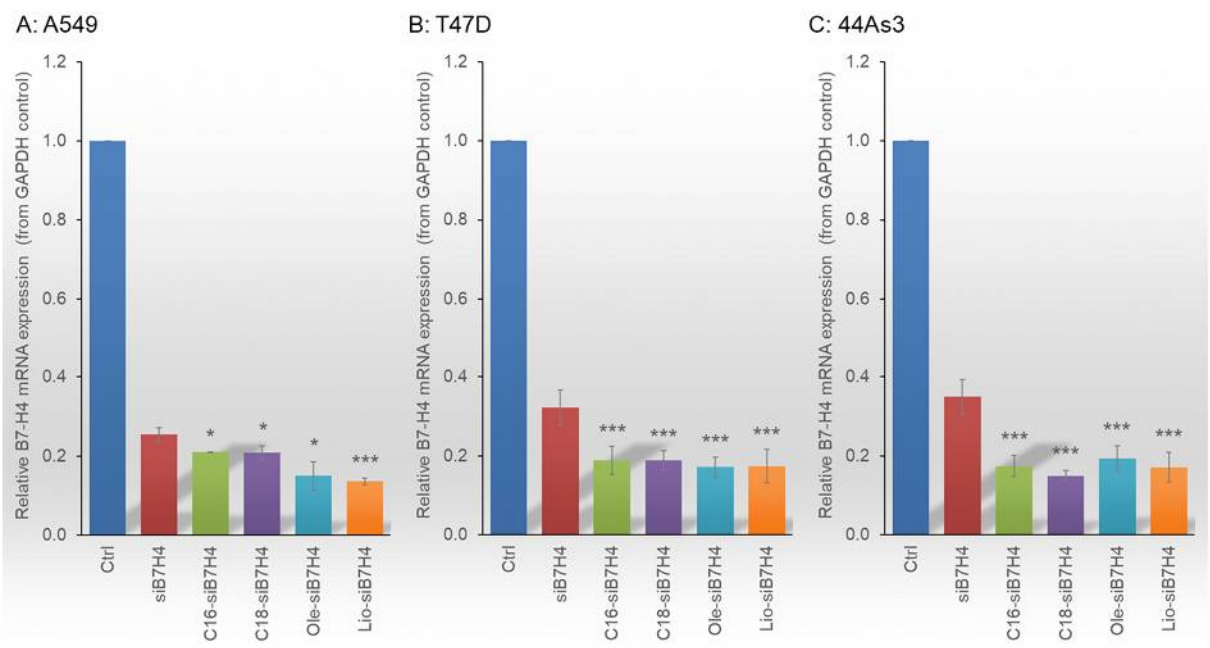

Figure S4. RNAi efficacy of siB7H4 and lipid-siB7H4s in A549 (A), T47D (B), and 44As3 (C) cells. siB7H4 and lipid-siB7H4s suppress B7-H4 expression by 70–90% in A549 cells, 70–80% in T47D cells, and 60–80% in 44As3 cells. In all cell lines, lipid-siB7H4s shows stronger RNAi effects than siB7H4. Data are presented as the mean ± SD of 3-6 independent experiments (* $P < 0.05$, ** $P < 0.01$, *** $P < 0.001$ vs. siB7H4; t-test). RNAi, RNA interference.

Original images of PAGE anlysis

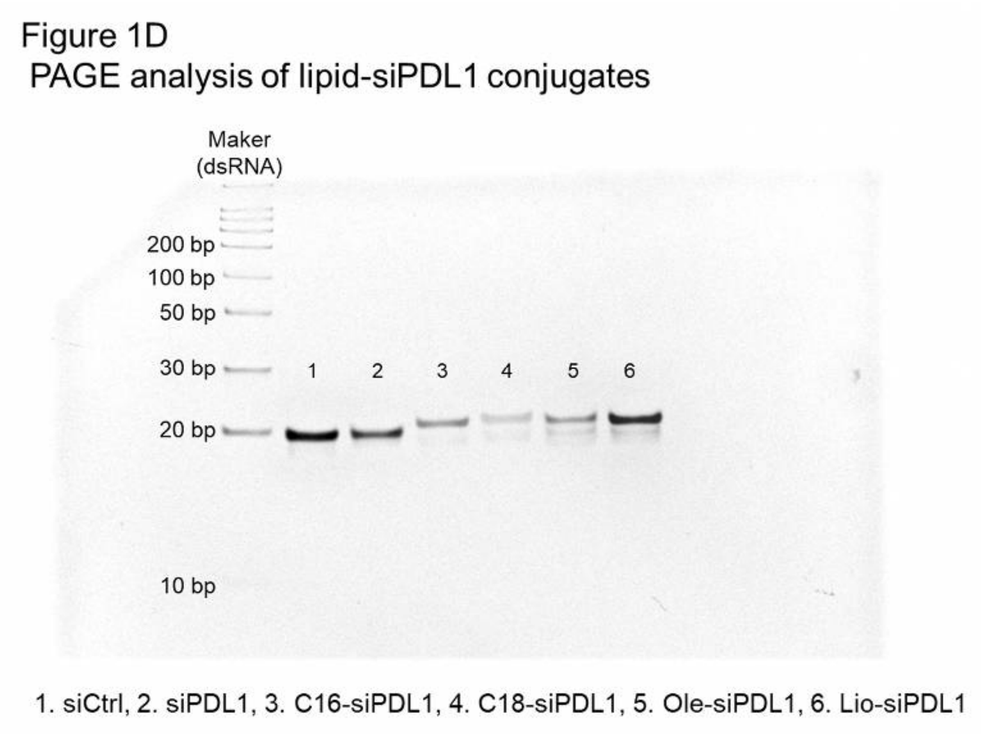

Original images of Western blots

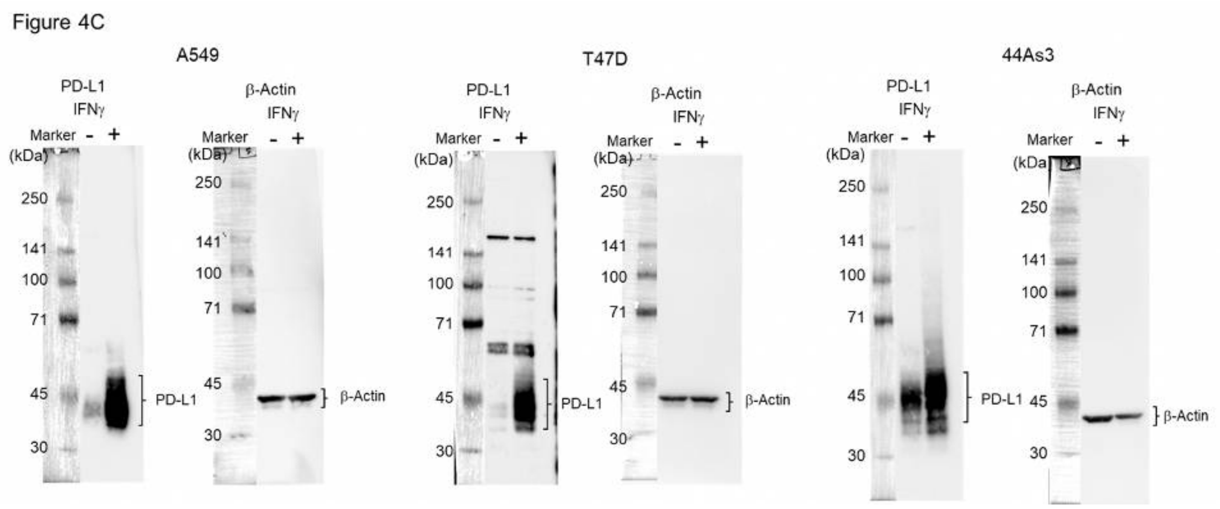

Figure 7 A. A549

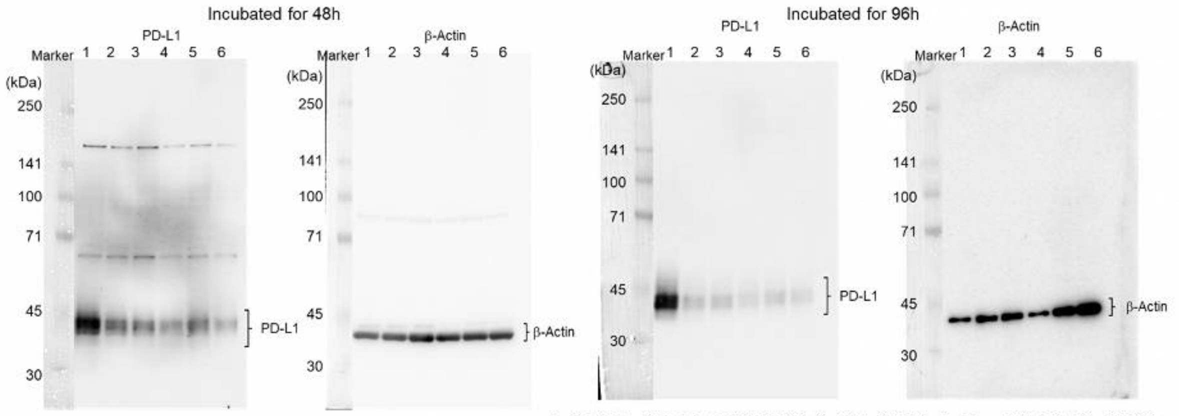

1. siCtrl, 2. siPDL1, 3. C16-siPDL1, 4. C18-siPDL1, 5. Ole-siPDL1, 6. Lio-siPDL1

Figure 7 B. T47D

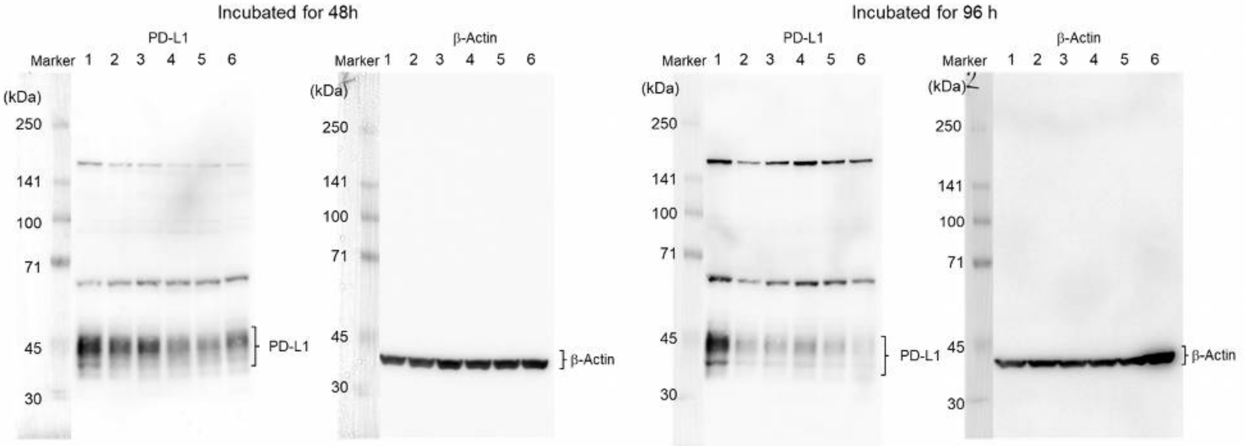

1. siCtrl, 2. siPDL1, 3. C16-siPDL1, 4. C18-siPDL1, 5. Ole-siPDL1, 6. Lio-siPDL1

Figure 7 C. 44AS3

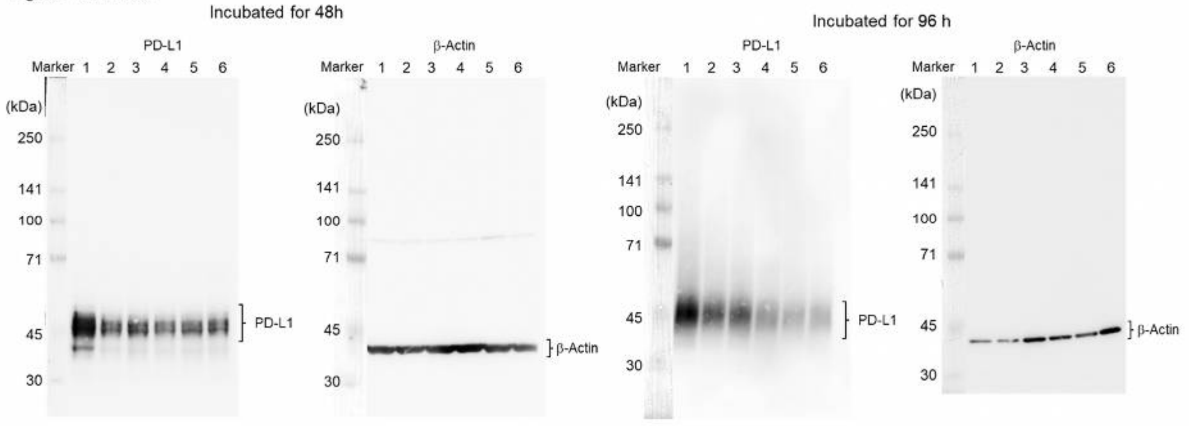

1. siCtrl, 2. siPDL1, 3. C16-siPDL1, 4. C18-siPDL1, 5. Ole-siPDL1, 6. Lio-siPDL1

- Original images of Western blots were created by overlaying of protein marker image detected by Epi-white light digitization and PD-L1 (or β-actin) protein image detected by chemiluminescence. All images of each marker and protein are on the same membrane.

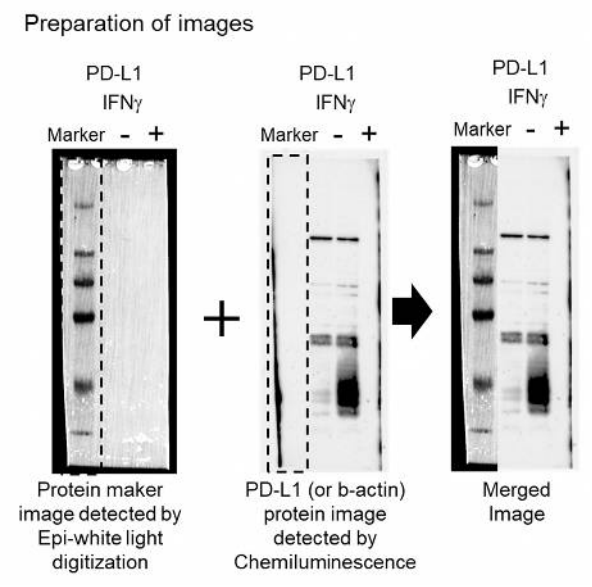

Information regarding the antibodies and protein maker used in this study can be found on the following websites

PD-L1 (E1L3N®) XP® Rabbit mAb (HRP Conjugate)

https://www.cellsignal.jp/products/antibody-conjugates/pd-l1-e1l3n-xp-rabbit-mab-hrp-conjugate/51296

Monoclonal Anti-β-Actin,

https://www.sigmaaldrich.com/deepweb/assets/sigmaaldrich/product/documents/255/782/a5441blot.pdf

DynaMarker® Protein MultiColor Stable II (Lot No: 006CD09)

https://bdl-biodynamics.com/wp-content/uploads/2024/03/DM660_006CD09En.pdf
